# Nil effects of μ-rhythm phase-dependent burst-rTMS on cortical excitability in humans: A resting-state EEG and TMS-EEG study

**DOI:** 10.1371/journal.pone.0208747

**Published:** 2018-12-07

**Authors:** Debora Desideri, Christoph Zrenner, Pedro Caldana Gordon, Ulf Ziemann, Paolo Belardinelli

**Affiliations:** 1 Department of Neurology & Stroke, and Hertie Institute for Clinical Brain Research, University of Tübingen, Tübingen, Germany; 2 Service of Interdisciplinary Neuromodulation, Laboratory of Neuroscience (LIM27) and National Institute of Biomarkers in Psychiatry (INBioN), Department and Institute of Psychiatry, Hospital das Clinicas HCFMUSP, Faculdade de Medicina, Universidade de São Paulo, São Paulo, Brazil; University of Bologna, ITALY

## Abstract

Repetitive transcranial magnetic stimulation (rTMS) can induce excitability changes of a stimulated brain area through synaptic plasticity mechanisms. High-frequency (100 Hz) triplets of rTMS synchronized to the negative but not the positive peak of the ongoing sensorimotor μ-rhythm isolated with the concurrently acquired electroencephalography (EEG) resulted in a reproducible long-term potentiation like increase of motor evoked potential (MEP) amplitude, an index of corticospinal excitability (Zrenner et al. 2018, Brain Stimul 11:374–389). Here, we analyzed the EEG and TMS-EEG data from (Zrenner et al., 2018) to investigate the effects of μ-rhythm-phase-dependent burst-rTMS on EEG-based measures of cortical excitability. We used resting-state EEG to assess μ- and β-power in the motor cortex ipsi- and contralateral to the stimulation, and single-pulse TMS-evoked and induced EEG responses in the stimulated motor cortex. We found that μ-rhythm-phase-dependent burst-rTMS did not significantly change any of these EEG measures, despite the presence of a significant differential and reproducible effect on MEP amplitude. We conclude that EEG measures of cortical excitability do not reflect corticospinal excitability as measured by MEP amplitude. Most likely this is explained by the fact that rTMS induces complex changes at the molecular and synaptic level towards both excitation and inhibition that cannot be differentiated at the macroscopic level by EEG.

## Introduction

Repetitive transcranial magnetic stimulation (rTMS) can be used to non-invasively modify excitability and connectivity of stimulated cortical areas [1]. In the motor cortex (M1), excitability changes can be assessed by comparing the responses to single-pulse TMS in a contralateral hand muscle (motor evoked potentials, MEPs) before and after rTMS. However, MEPs represent an indirect measure of cortical and corticospinal excitability. It is believed that rTMS-induced changes in MEP amplitude most likely originate at the level of the motor cortex (for review see [2]), therefore rTMS would be expected to produce lasting changes in cortical excitability and connectivity. Electroencephalography (EEG) is used to measure electrical activity of the brain [3] and in combination with TMS (TMS-EEG) provides access to a more direct measure of cortical excitability and connectivity. Cortical responses to TMS are expressed in the EEG in a series of evoked potentials (TEPs) [4–6] as well as in a modulation of spontaneous oscillatory activity [7–10]. Previous studies showed that rTMS induced LTP-/LTD-like plastic changes can be successfully measured with TMS-EEG at a macroscopic scale. For example, Esser and colleagues [11] reported an increase both in MEP size and in the global mean field power (a compressed representation of the total TMS-evoked response) in the area of premotor cortex after 5 Hz rTMS of the left M1. Also, in [12], 1 Hz rTMS decreased MEPs and produced a significant increase in the TEP components peaking at around 60 ms and 100 ms after TMS. High-frequency rTMS of the left M1 has also been found to affect oscillatory activity in the α and β bands, both at the stimulation site and in interconnected cortical areas [13, 14]. In a previous study of our group [15], we have shown that the negative vs. positive peak of the ongoing sensorimotor oscillation in the 8–12 Hz frequency band (μ-oscillation) represents a state of higher excitability of the motor system, where the dendritic trees of pyramidal neurons receive mainly excitatory inputs and are closer to firing threshold. Results of *in vitro* studies [16–19] showed that the direction of synaptic plasticity critically depends on the coupling between stimulus and the state of neuronal population receiving it. On this basis, we have investigated the after-effects of high-frequency (100 Hz) triplets of rTMS delivered to M1, synchronized to the instantaneous phase of the ongoing sensorimotor oscillation in the 8–12 Hz frequency band (μ-oscillation) and demonstrated that when rTMS is applied at the negative peak of the μ-oscillation MEP size increases for more than 30 minutes after the intervention, compatible with long-term potentiation (LTP)-like plasticity, while no significant changes in MEP size are observed when rTMS is applied at the positive peak of the μ-oscillation [15]. In this study, we sought to investigate whether μ-rhythm phase-dependent rTMS produces similar changes in cortical excitability that are expressed in the spontaneous oscillatory activity of M1 ipsi- and contralateral to the stimulation in the resting-state EEG, and/or in the TEPs and in the oscillations induced by TMS over the stimulated M1. Oscillatory activity, both spontaneous and induced by TMS, has been analyzed in the μ- and β-bands, as these are the typical and dominant oscillations in the sensorimotor system [20].

## Materials and methods

The EEG data used in this study have been collected in [15].

### Subjects

**Fig 1:**
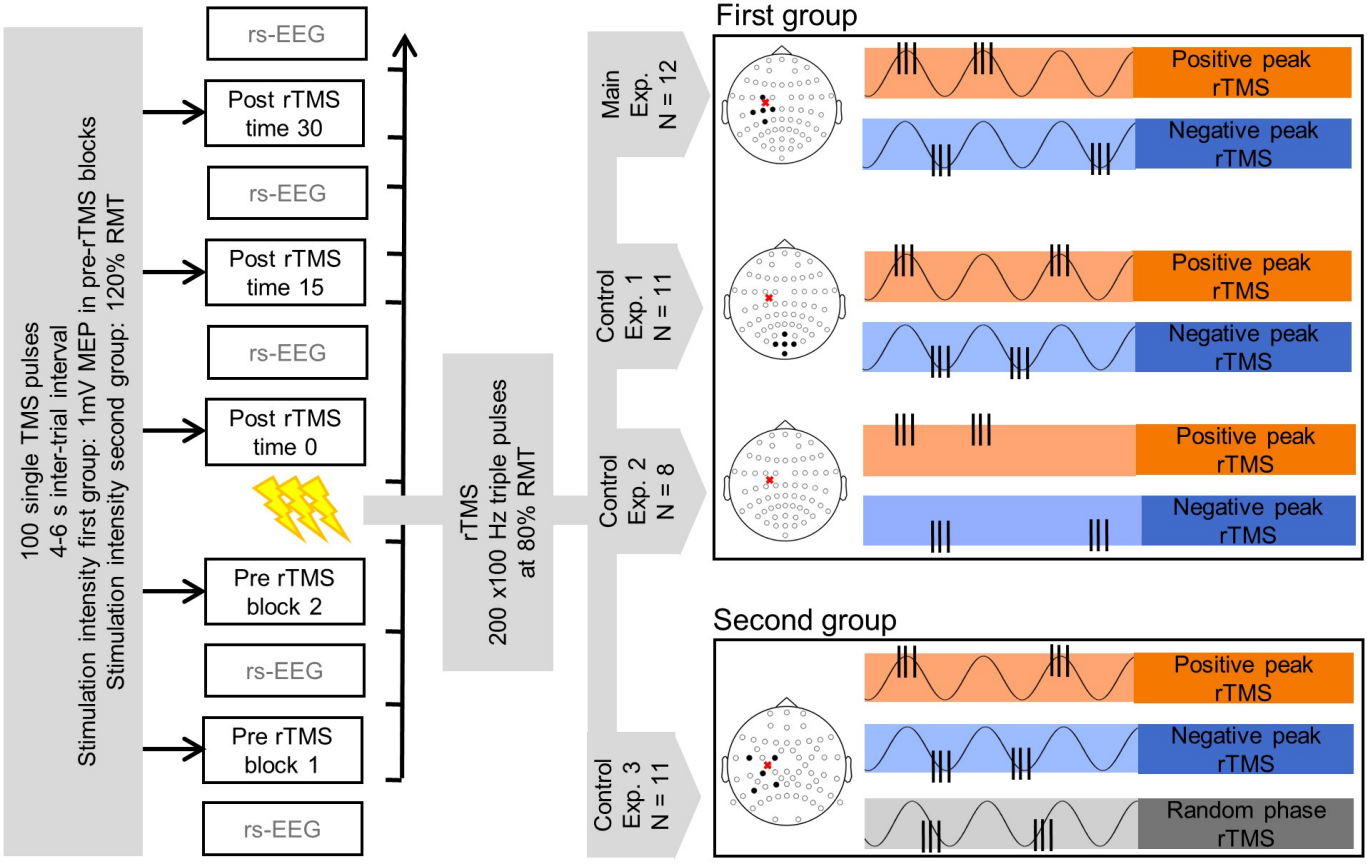
Experimental design and sessions. Left side: blocks of 100 single TMS pulses were used to test effects of different rTMS interventions on cortical and corticospinal measurements. Two pre-rTMS test blocks were acquired 15 minutes apart and three test blocks were acquired at 0,15 and 30 minutes after rTMS. Resting state EEG periods of ca. 4–5 minutes preceded the test blocks before rTMS and followed them after rTMS. The rTMS intervention consisted of 200 bursts of triple pulses TMS with an inter-pulse interval of 10 ms (100 Hz triplets) delivered at 80% RMT. The mean triplet repetition rate ± 1 s.d. was 1.01±0.12 Hz and 1.04±0.08 Hz for the first and second group of participants, respectively. Right side: sessions differed from one another in the brain state used to trigger the rTMS triplets. TMS was applied to the left motor cortex, as indicated by the red cross in the schematic picture of the participants’ head. Circles represent the positions of the EEG electrodes, filled black circles highlight the EEG electrodes used to isolate the brain signal of interest to trigger rTMS. In the first group of participants, rTMS was triggered by the phase (positive or negative peak) of the left sensorimotor μ-rhythm (Main Experiment, 12 subjects), by the phase (positive or negative peak) of the endogenous occipital α-oscillation (Control Experiment 1, 11 subjects) or by the identical rTMS sequence of the Main Experiment (Control Experiment 2, 8 subjects). In the second group of participants, rTMS was triggered by the phase (positive peak, negative peak or random) of the left sensorimotor μ-rhythm (Control Experiment 3, 11 subjects). The order of the sessions was pseudo-randomized for the first group of participants (with Control Experiment 2 sessions always following Main Experiment sessions) and fully randomized for the second group of participants. For a given participant, consecutive sessions were performed at least 3 days apart.

The study protocol was approved by the local ethics committee of the medical faculty of the University of Tübingen (protocol 716/2014BO2). Experiments were conducted in accordance with the declaration of Helsinki and the current TMS safety guidelines [21]. Two groups of volunteers free of medications and without history of neurological or psychiatric disorders took part in the study, after giving written informed consent. Twelve right-handed male participants (age range 20–48 years, mean age±s.d. 26.5±7.5 years, Edinburgh Handedness inventory laterality score 62±21) were tested in the first series of experiments, while eleven right-handed participants (9 females, age range 21–32 years, mean age±s.d. 25.4±3.7 years, Edinburgh Handedness inventory laterality score 91±13) were tested in the second series of experiments (see below *Experimental design* and Fig 1). All included subjects exhibited a spectral power in the μ-band (8–12 Hz) > 25% of the power in the 1–80 Hz power spectrum of the current scalp density (CSD) signal of the C3 EEG electrode. This ensured a sufficient signal-to-noise ratio for a reliable real-time estimation of the instantaneous phase of the sensorimotor μ-oscillation [15]. Moreover, resting motor threshold (RMT) of the right abductor pollicis brevis (APB) or the first dorsal interosseous (FDI) muscle had to be ≤ 60% and ≤ 67.5% of maximum stimulator output for the first and second group, respectively. This guaranteed that for each participant 80% RMT did not exceed the maximum intensity at which the stimulators used in the two series of experiments (see below *Experimental set-up & procedure*) could generate 100 Hz rTMS pulses (48% and 54%, respectively). When RMT was comparable for APB and FDI, APB was used as target muscle, otherwise the muscle with lower RMT was chosen to increase the chance of a subject being included in the study. APB was the target muscle for 3/12 subjects in the first group and for 6/11 subjects in the second group. For each subject, the target muscle was kept consistent across different experimental sessions.

### Experimental set-up & procedure

Participants were seated in an armchair with their hands relaxed and looking at a fixation cross while TMS pulses were delivered to the left M1. In the first series of experiments the set-up consisted of an air cooled TMS coil (Magstim 70 mm Double Air Film Coil, Magstim, Ltd, UK) connected to a high-frequency magnetic stimulator (Magstim Super Rapid Plus, Magstim Ltd, UK) delivering biphasic single cosine cycle pulses with 400 μs period. In the second series of experiments a passively cooled TMS double coil (PMD70-pCool, 70 mm winding diameter, MAG & More, Germany) connected to a high frequency magnetic stimulator (research 100, MAG & More, Germany) delivering biphasic single cosine cycle pulses with 160 μs period was used instead. Motor evoked potentials (MEPs) were recorded from the right APB and FDI muscles in a bipolar belly-tendon montage through electromyography (EMG, 5 kHz sampling rate, 0.16 Hz -1.25 kHz bandpass filter) adhesive hydrogel electrodes (Kendall, Covidien). RMT was defined according to the standard relative frequency method [22] as the minimum stimulation intensity that produced MEPs > 50 μV in the target muscle in 5/10 consecutive trials. The motor "hotspot" was defined as the coil position and orientation eliciting maximum MEP amplitudes, using a slightly suprathreshold stimulation intensity [22]. The stimulating coil was oriented orthogonally to the central sulcus, so that the second phase of the biphasic pulse induced a lateral-posterior to medial-anterior electric field in the brain. Coil position was kept constant relative to the participant’s head using a stereoscopic neuronavigation system (Localite GmbH, Sankt Augustin, Germany). The EEG signal was recorded from 64 channels inserted in a TMS compatible Ag/AgCl sintered ring electrode cap (EasyCap GmbH, Germany). In the first series of experiments, the EEG channels were arranged according to the International 10–20 system and FCz and AFz were used as reference and ground electrode, respectively. In the second series of experiments, a customized layout based on the International 10–20 system, but with higher electrode density over sensorimotor cortex was used and FCz and PPO1h were taken as reference and ground electrode, respectively. The impedance at the interface skin-EEG electrodes was < 5 kΩ throughout. A 24-bit 80-channel biosignal amplifier was used for EEG and EMG recordings (NeurOne Tesla with Analog Real-time Out Option, Bittium Biosignals Ltd., Finland). Data were acquired at 5 kHz sampling rate in AC mode for the first series of experiments (band pass filter 0.16 Hz—1.25 kHz) and in DC mode for the second series of experiments (1.25 kHz low-pass anti-aliasing filter) with a head-stage sample rate of 80 kHz. A real-time system implemented as a Simulink Real-Time model (Mathworks Ltd, USA, R2015a), executed on a dedicated xPC Target PC running the Simulink Real-Time operating system (DFI-ACP CL630-CRM mainboard) was used for a real-time analysis of the EEG signal and for triggering rTMS bursts according to the instantaneous phase of oscillatory EEG activity (for details [15]). White noise at individually adjusted loudness was applied through ear phones to mask the TMS click and minimize TMS-evoked auditory potentials [23, 24].

### Experimental design

The experimental design is schematically represented in Fig 1. Each experimental session consisted of 2 pre-rTMS and 3 post-rTMS blocks of 100 suprathreshold single-pulse TMS trials and of 2 pre-rTMS and 3 post-rTMS periods of approximately 4–5 minutes resting-state EEG (rsEEG) recording with eyes open. The 2 pre-rTMS blocks started 15 minutes apart, as well as the 3 post-rTMS blocks, while the last pre- and first post-rTMS blocks were separated only by the duration of the rTMS intervention. The rsEEG recordings preceded the single-pulse TMS blocks in the pre-rTMS and followed them in the post-rTMS measurements. The rTMS intervention consisted of 200 bursts of 100 Hz TMS bursts delivered at 80% RMT. In each session, triplets were triggered by a different pre-defined brain state.

In the first series of experiments the following brain states were used to trigger the rTMS triplets: positive peak or negative peak of the local μ-rhythm (Main Experiment), positive peak or negative peak of distal occipital α-rhythm (Control Experiment 1), replay of positive peak or negative peak μ-rhythm rTMS sequence from Main Experiment independent of ongoing μ-rhythm (Control Experiment 2). Local μ-rhythm originating in the left sensorimotor cortex was isolated at sensor level with a spatial filter [25] centered on the electrode C3 and using the four adjacent electrodes FC3, C1, CP3, C5. A similar filter centered on the electrode Oz and using the neighbor electrodes POz, O1, O2, Iz was used to isolate the occipital α-rhythm. The Main Experiment investigated the effects of sensorimotor μ-phase on corticospinal plasticity. Control Experiment 1 served to demonstrate that the phase-dependent effects on corticospinal plasticity observed in the Main Experiment were produced only when a locally generated oscillation is used to trigger the rTMS triplets. Control Experiment 2 was used to show that the results in the Main Experiment depended on the synchronization between stimulus and brain state and were not caused by the temporal properties of the rTMS sequence.

In the second series of experiment the following brain states were used to trigger the rTMS triplets: positive peak, negative peak or random phase of the local μ-rhythm (Control Experiment 3). Local μ-rhythm was isolated with a slightly different spatial filter centered on the electrode C3 and using the neighbor electrodes FC1, FC5, CP1, CP5. This different spatial filter was used to be able to record oscillatory activity also from an off-center EEG source of the μ-rhythm. Control Experiment 3 was designed to validate, generalize and extend the results of the Main Experiment.

In the brain state-triggered sessions a power threshold was used to ensure that triplets were triggered by physiological signal instead of filtered noise. The power threshold was set manually and chosen to ensure an average inter-burst interval of approximately 1 s [15]. The mean triplet repetition rate ± 1 s.d. was 1.01±0.12 Hz and 1.04±0.08 Hz for the first and second group of participants, respectively. For a given participant, consecutive sessions were performed at least 3 days apart and the order of the sessions was pseudo-randomized for the first group (with Control Experiment 2 sessions always following Main Experiment sessions) and fully randomized for the second group of participants.

### Data analysis

Data processing and analysis were performed using customized analysis scripts on MATLAB R2018a and the Fieldtrip open source MATLAB toolbox [26].

#### Resting-state EEG

The rsEEG periods were segmented in epochs of 2 s, down-sampled to 500 Hz and filtered with a 1–80 Hz bandpass filter (zero-phase Butterworth, 3^rd^ order) and a 49–51 Hz notch filter (zero-phase Butterworth, 3^rd^ order). Epochs containing major artifacts as well as noisy EEG channels were removed upon visual inspection (percentage of removed epochs: 9.7±5.9%, number of removed channels: 2±2, mean±s.d.). Then, independent component analysis (ICA) based on FastICA algorithm [27, 28] was applied to the data. ICA components representing biological (eye blinks and movements, persistent muscle activity) or electrical artifacts were removed based on their topography, single-trial time-course and power spectrum (mean±s.d. 22±6). Discarded channels were spline-interpolated using the signal of the neighbor channels [29]. CSD signals [29] of the EEG channels C3 and C4, chosen as representative of the left and right sensorimotor cortices, respectively, were computed and their power spectra were estimated based on the fast Fourier transform (FFT) algorithm. For each subject and channel, individual μ- and β-bands were defined as 2 Hz wide bands centered at the maximum peak in the 5–15 Hz and 15–30 Hz ranges of the average power spectrum, respectively. Finally, for each subject, rsEEG period and session, C3 and C4 μ- and β-powers were defined as the power in the individual C3 and C4 μ- and β-bands and used for statistical analysis (see below, *Statistics*).

#### TMS-EEG evoked potentials

**Fig 2:**
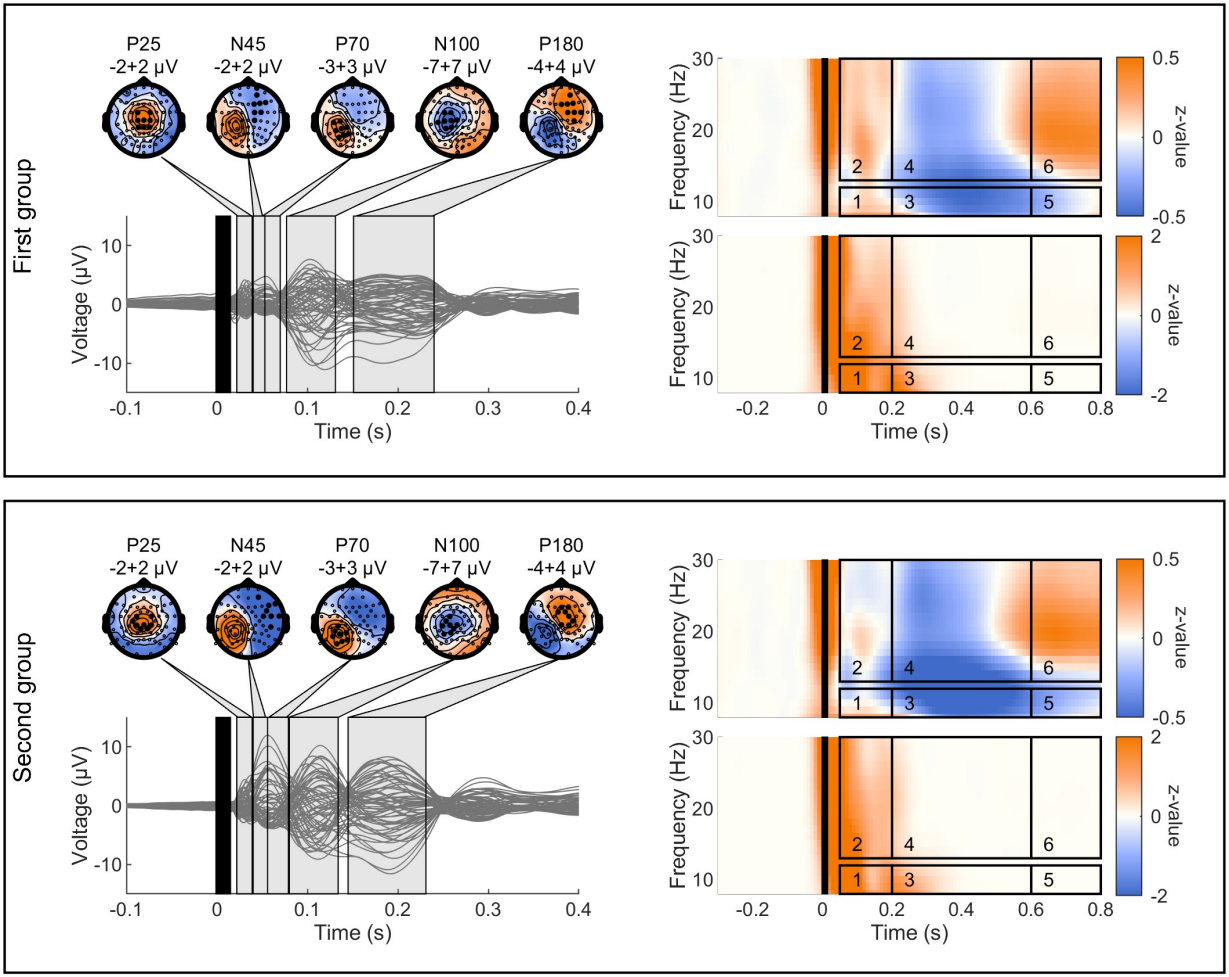
Selection of TEPs TOIs and channels and induced and evoked oscillations ROIs. On the left, single EEG channel traces and topographical plots resulting from the average across all subjects, test blocks and sessions for the first (top) and second (bottom) group of subjects. TEPs peaks are named according to their approximate latency after TMS application. Topographical distributions are obtained averaging the signal in the grey-shaded time windows. The maximum and minimum voltages are reported in μV below the relative topographies. Black filled dots in the topographies indicate the channels where the corresponding TEP peak was maximally expressed and that were used for calculation of LMFP in the relative TOI. On the right side, TFRs of induced (upper part) and evoked (lower part) oscillations on channel C3 averaged across all subjects, test blocks and sessions for the first (top) and second (bottom) group of subjects. Time-frequency ROIs used for the analysis are enclosed by the black rectangles. In all plots, time windows highlighted in black indicate signal affected by TMS-related artifacts that was removed and interpolated.

The single-pulse TMS trials were segmented in epochs from 0.5 s before the TMS marker to 1 s after the TMS marker. Data from 1 ms before to 15 ms after the marker, where high amplitude TMS artifacts occur, were removed and cubic interpolated. Epochs were then centered and visually inspected (percentage of removed epochs: 4.0 ± 3.4%, number of removed channels: 3±2, mean±s.d.). Then, the same ICA used for the rsEEG data was applied to the TMS-EEG data. Data underwent ICA twice, in a two-step procedure as proposed in [30]. In the first step, only components representing high-amplitude TMS-related artifacts were removed (mean±s.d. 7±4). Then, data were filtered with a 1–80 Hz bandpass filter (zero-phase Butterworth, 3^rd^ order) and a 49–51 Hz notch filter (zero-phase Butterworth, 3^rd^ order), down-sampled to 1000 Hz and ICA was again applied to the data. Components representing biological (eye blinks and movements, persistent muscle activity), electrical or smaller amplitude TMS-related artifacts were removed (mean±s.d. 22±6). Then, channels discarded during the visual inspection of the data were spline-interpolated [29] and data were re-referenced to the average reference signal. Epochs were lowpass filtered (45 Hz, zero-phase Butterworth, 3^rd^ order) and averaged per block. Five non-overlapping time windows of interest (TOIs) were *a priori* defined based on the group average TEPs across subjects, blocks and sessions. The pre-selected TOIs were approximately centered around the latencies of the typical motor cortex TEP peaks P25, N45, P70, N100 and P180 [4, 6, 31]. Specifically, beginning and end of the TOIs were set at 22–39 ms, 40–53 ms, 53–70 ms, 77–131 ms, 151–240 ms after the TMS pulse, respectively, for the first group of subjects, and at 22–39 ms, 40–56 ms, 56–79 ms, 80–134 ms, 145–231 ms for the second group of subjects. Moreover, for each TOI, eight channels of interest were defined as the channels with maximum voltage for P25, P70 and P180 and the channels with minimum voltage for N45 and N100 (first group: P25 –Cz, FC1, C1, C2, FC2, CP2, CPz, CP1, N45 –F2, FC2, F4, AF4, C2, FC4, Fp2, F6, P70 –CP3, C5, C3, P5, P1, CP5, P3, CP1, N100 –C1, C3, CP3, FC1, CP1, FC3, Cz, C5, P180 –Fz, F2, FC2, F4, FC4, AF4, C4, FC6; second group: P25 –Cz, C1, CCP1h, CCP2h, C2, FCC3h, FC1, FC2, N45 –FFC6h, FT8, C6, F6, FC4, FC6, AF4, F8, P70 –CP3, CCP5h, P5, P1, P3, CP5, C3, CP1, N100 –C1, FCC3h, C3, Cz, FC1, CCP1h, CP1, CP3, P180 –FC2, FFC2h, FCC4h, C2, FFC1h, Cz, Fz, F2). Selected TOIs and channels for each TOI can be seen in Fig 2. Information about TMS-evoked cortical activation was then compressed computing the local mean field power (LMFP, [12, 32–34]) over the selected channels in the corresponding TOI. For each TOI, subject, test block and session the area under the obtained LMFP was used for statistical analysis (see below, *Statistics*).

#### TMS-EEG induced oscillations

A time-frequency representations (TFRs) of the TMS-EEG data can uncover responses to TMS that are not time- locked to the onset of the pulse and are expressed as changes in spontaneous oscillatory activity [7–10, 35]. We have focused on the CSD signal of the C3 channel and have isolated the non-time-locked, i.e. induced, activity, in the time-domain by subtracting the average evoked response from each single trial [10, 35, 36]. Subsequently, we have calculated the TFRs convolving single trials with complex Morlet wavelets [37] in the frequency range from 6 to 45 Hz in steps of 1 Hz and shifting the center of the wavelet in steps of 10 ms in the time window -500 ms to 1000 ms relative to TMS application. The length of the wavelets linearly increased from 2 cycles at 6 Hz to 9 cycles at 45 Hz [35, 38]. TFRs of power were obtained taking the squared absolute values of the complex time series resulting from the wavelet transformation. These were then trial-wise z-transformed based on the mean and standard deviation of the full-length trial as described in [39] and baseline corrected subtracting the mean value (over time) of the baseline period (from 300 ms to 100 ms before TMS), to ensure that the average pre-TMS values did not differ from zero and that z-values could be interpreted as a modulation of the pre-TMS oscillatory activity. Finally, TFRs were averaged per block and trimmed to remove the time points where no time-frequency values could be calculated (from -500 to -333 ms and from 833 to 1000 ms with respect to the TMS marker in the data, corresponding to 1 cycle of the 6 Hz oscillation at the beginning and end of the epoch). Based on previous literature [10, 40], six non-overlapping time-frequency regions of interest (ROIs) were *a priori* defined (Fig 2). For each subject, block and session, time-frequency values were averaged in the pre-defined ROIs (ROI1–8–12 Hz, 50–200 ms, ROI2–13–30 Hz, 50–200 ms, ROI3–8–12 Hz, 200–600 ms, ROI4–13–30 Hz, 200–600 ms, ROI5–8–12 Hz, 600–800 ms, ROI6- 13–30 Hz, 600–800 ms) and used for statistical analysis (see below, *Statistics*).

*TMS-EEG evoked oscillations*: Local TMS-EEG evoked oscillatory activity [6, 7, 41, 42] was obtained averaging across trials the CSD signal of the C3 channel in each block and calculating the TFRs thereof with complex Morlet wavelets as described in the previous paragraph. For each subject, block and session, time-frequency values were averaged in the same six pre-defined ROIs of the induced activity (Fig 2) and used for statistical analysis (see below, *Statistics*).

#### Statistics

For each group of participants and for each investigated parameter, repeated measures analyses of variance (rmANOVAs) were run on the raw, i.e. not normalized, pre-rTMS data to demonstrate that there was no effect of TIME (2 levels, i.e. block 1 and block 2), SESSION (6 levels for first group of participants, 3 levels for second group of participants) or the interaction SESSION*TIME. For this analysis, the rsEEG data and LMFP data were ln-transformed to ensure normality. Then for each experiment, i.e. Main Experiment, Control Experiment 1, Control Experiment 2 and Control Experiment 3, and for each investigated parameter, rmANOVAs with the within–subject effects of PHASE (2 levels, i.e. positive and negative peak, for Main Experiment, Control Experiment 1 and Control Experiment 2, 3 levels, i.e. positive peak, negative peak and random phase, for Control experiment 3) and TIME (3 levels, i.e. time 0, time 15, time 30), were performed on normalized data. For the normalization, the rsEEG and LMFP data were divided by the average of the pre-rTMS blocks, while the average of the pre-rTMS blocks was subtracted from the TFRs data. Sphericity in all rmANOVAs was tested using Mauchly’s test and, whenever it was violated, the Greenhouse-Geisser correction was applied. Post-hoc paired two-tailed t-tests were applied in case of a significant main effects or interactions. For all tests, the Lilliefors test was used to verify normal distribution. The significance level was p<0.05. Bonferroni’s correction was used for all *post hoc* analyses following rmANOVA to correct for multiple comparisons.

## Results

### Effect of rTMS on resting-state EEG μ- and β-power

**Fig 3:**
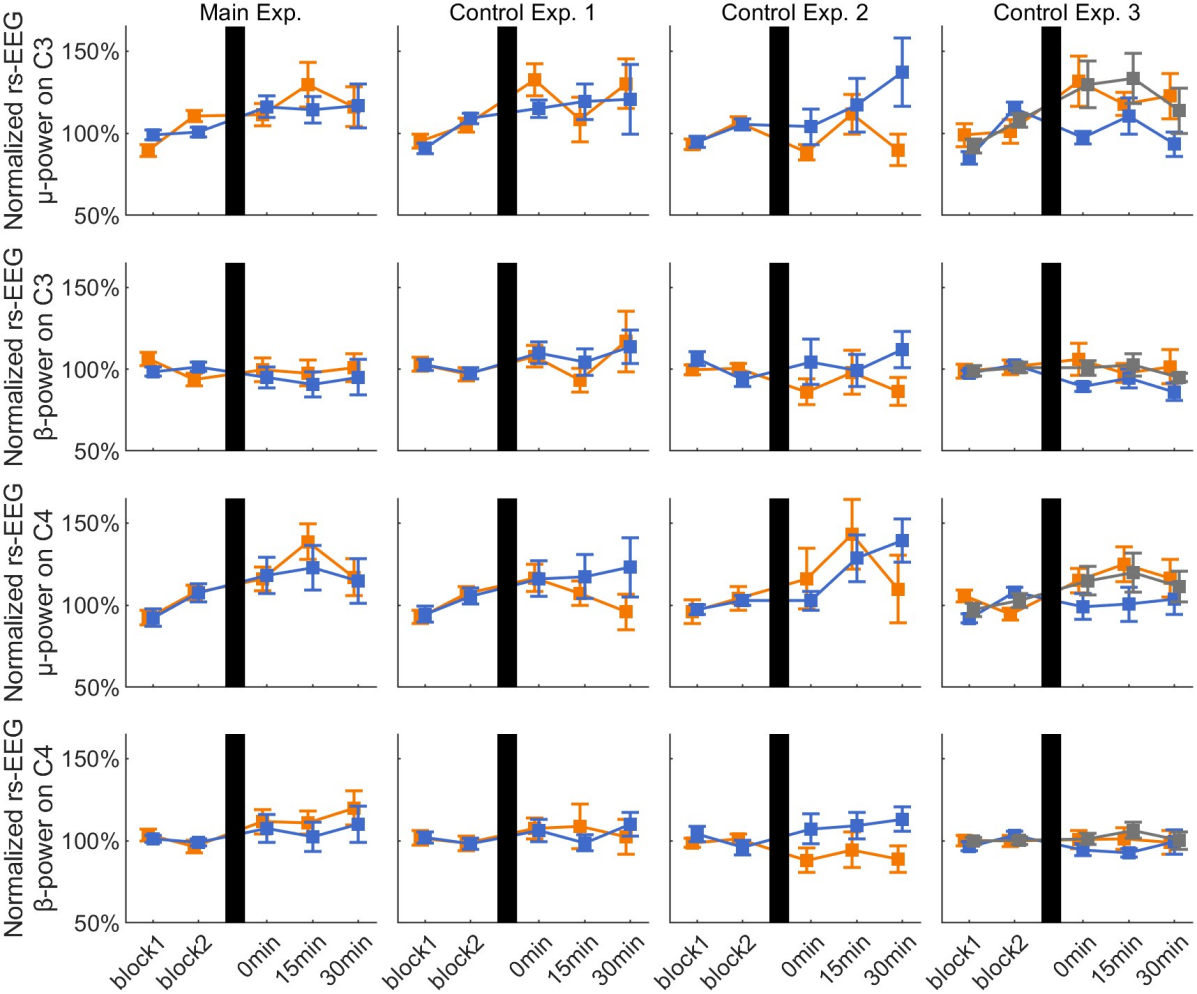
Resting-state EEG μ- and β-power on C3 and C4. Time course of group average ± 1 s.e.m. resting-state EEG μ- and β-power on channels C3 (upper rows) and C4 (lower rows), for the positive (orange lines), negative (blue lines) and random (gray lines) phase triggered conditions in each experiment. Data are normalized to the average of the pre-rTMS blocks. In all plots, vertical black bars indicate the rTMS intervention.

Fig 3 shows the normalized μ- and β-power on C3 and C4 in the rsEEG periods for all the experimental sessions. C3 μ-power showed a significant effect of TIME in Control Exp. 2 (F_2,14_ = 4.825, p = 0.025), while C3 β-power showed a significant interaction PHASE*TIME in Control Exp. 2 (F_2,14_ = 5.601, p = 0.029). In both cases, all *post hoc* pairwise comparisons were not significant (all p > 0.05).

### Effect of rTMS on TEP LMFPs

**Fig 4:**
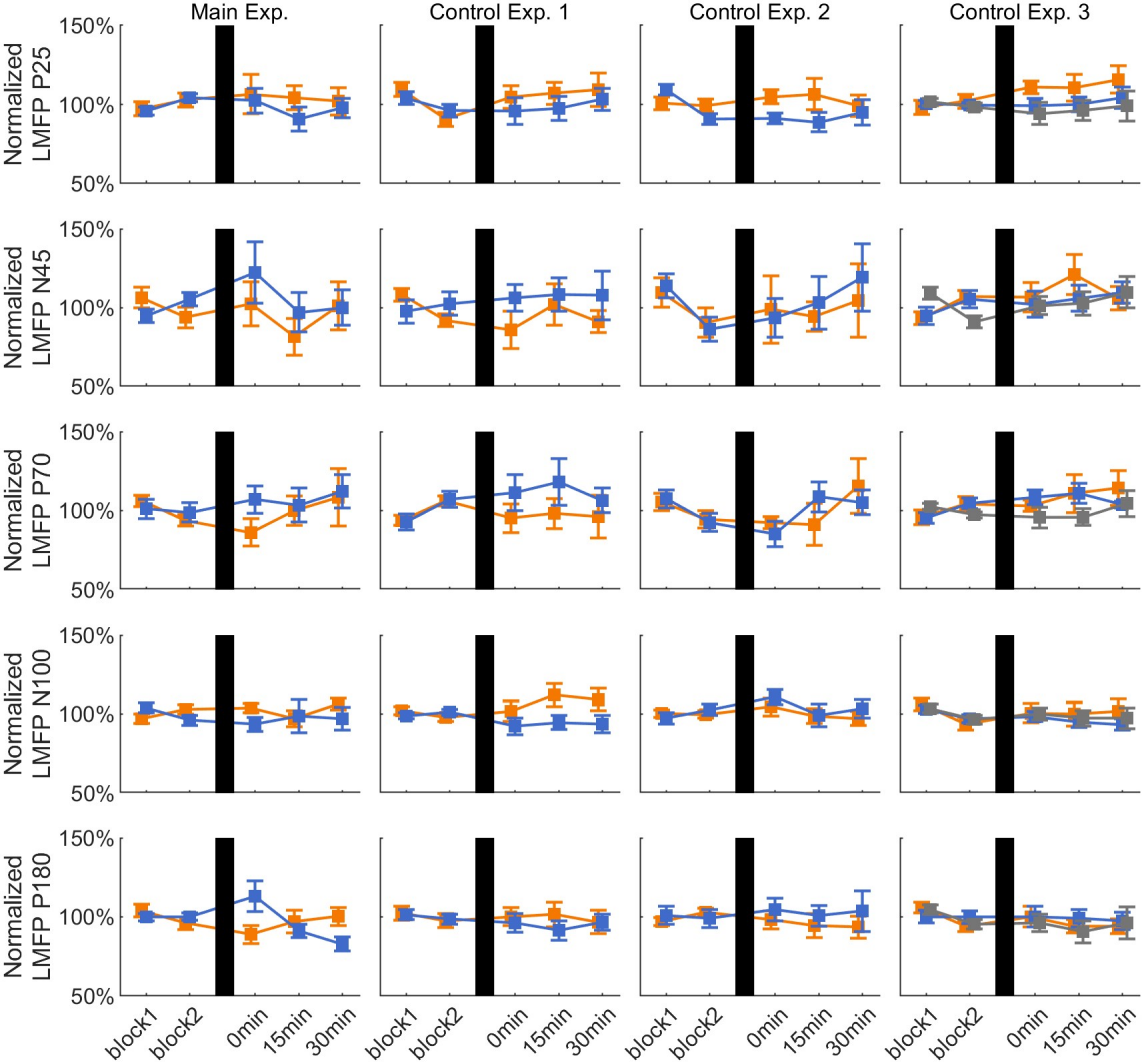
LMFP of TEPs. Time course of group average ± 1 s.e.m. of LMFP of TEP components (P25, N45, P70, N100, P180) for the positive (orange lines), negative (blue lines) and random (gray lines) phase triggered conditions in each experiment. Data are normalized to the average of the pre-rTMS blocks. In all plots, vertical black bars indicate the rTMS intervention.

Fig 4 shows the normalized LMFP of the different TEP components for all the experimental sessions. In the Main Experiment, LMFP data showed a significant interaction PHASE*TIME for the P180 TEP component (F_2,22_ = 8.734, p = 0.006). *Post hoc* pairwise comparisons demonstrated a decrease of the P180 LMFP 30 min after rTMS in the negative peak condition (p = 0.027) compared to the average pre-rTMS value. In Control Experiment 1, there was a significant effect of PHASE for the N100 TEP (F_1,10_ = 8.649, p = 0.015). Pairwise comparisons revealed an increased N100 LMFP in the positive peak vs. negative peak condition (p = 0.045).

### Effect of rTMS on local TMS-induced oscillations

**Fig 5:**
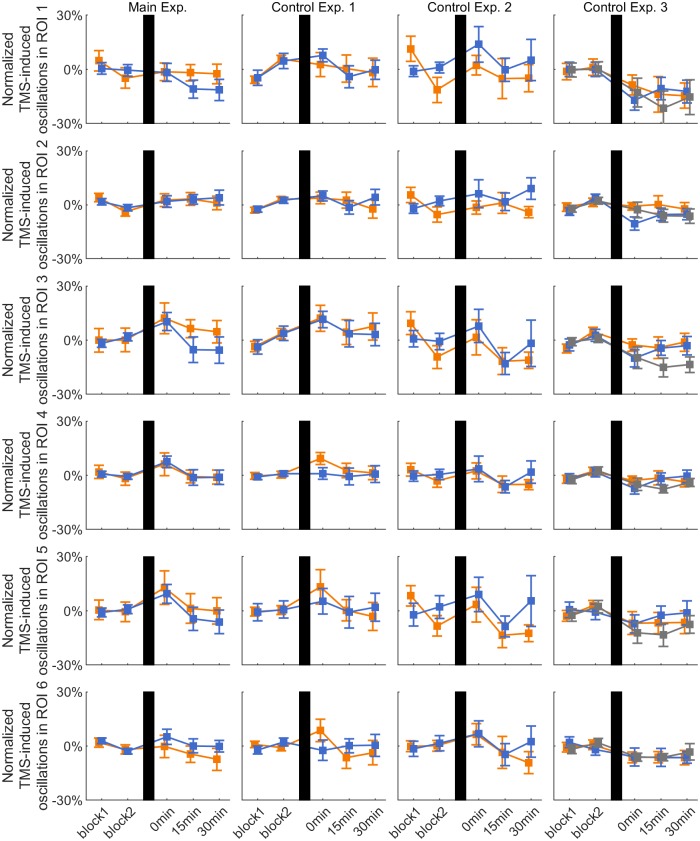
TMS-induced oscillations on C3. Time course of group average ± 1 s.e.m. of TMS-induced oscillations on channel C3 in the six *a priori* defined ROIs for the positive (orange lines), negative (blue lines) and random (gray lines) phase triggered conditions in each experiment. Data are normalized to the average of the pre-rTMS blocks. In all plots, vertical black bars indicate the rTMS intervention.

Fig 5 shows the normalized TMS-induced oscillations on C3 in the six *a priori* defined ROIs for all the experimental sessions. TFRs data showed a significant effect of PHASE for ROI 3 (F_2,20_ = 3.565, p = 0.047) in Control Experiment 3 and a significant effect of TIME for ROI 3 (F_2,14_ = 4.844, p = 0.0275) and ROI 5 (F_2,14_ = 5.167, p = 0.021) and ROI 6 (F_2,14_ = 4.509, p = 0.031) in Control Experiment 2. Post hoc pairwise comparisons were all non-significant after Bonferroni correction (all p > 0.05).

#### Effect of rTMS on local TMS-evoked oscillations

**Fig 6:**
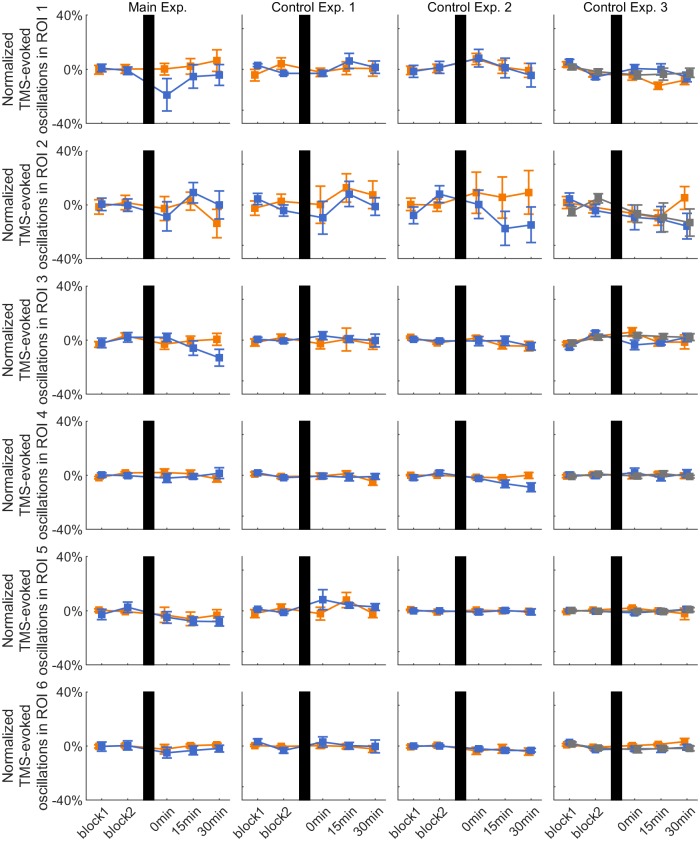
TMS-evoked oscillations on C3. Time course of group average ± 1 s.e.m. of TMS-evoked oscillations on channel C3 in the six *a priori* defined ROIs for the positive (orange lines), negative (blue lines) and random (gray lines) phase triggered conditions in each experiment. Data are normalized to the average of the pre-rTMS blocks. In all plots, vertical black bars indicate the rTMS intervention.

Fig 6 shows the normalized TMS-evoked oscillations on C3 in the six *a priori* defined ROIs for all the experimental sessions. TFRs data showed no significant main effect of PHASE, TIME or of the interaction PHASE*TIME in any of the ROIs (all p>0.05).

## Discussion

We have investigated here the effects of μ-rhythm phase-dependent rTMS on several EEG measures of cortical excitability, i.e. resting-state EEG μ- and β-power, TEP LMFPs and TMS-induced and -evoked oscillatory activity. In line with previous results from our group on corticospinal plasticity [15], we expected LTP-like changes of these parameters when rTMS was applied at the negative peak of the local μ-oscillation, but none of the EEG measures was consistently and differentially modulated by rTMS when triggered on the negative vs. positive peak of the μ-rhythm. At a first glance, our results may seem counterintuitive, as a clear and reproducible change in MEP size, i.e. the long-term increase with synchronization of rTMS to the negative peak of the μ-rhythm, was not paralleled by similar changes in the investigated EEG measures. There is sufficient evidence that high-frequency rTMS-induced long-term increases in MEP amplitude occur at the level of cortex, as demonstrated, for example, by a reduced paired-pulse inhibition [43] and an increased regional cerebral metabolic rate of glucose [44] in the stimulated motor cortex. This notion is also supported by studies in animals showing that rTMS can modulate protein expression [45, 46], neurotransmitter and neuromodulator release and turnover [47, 48], as well as neuronal spiking [46]. However, several TMS-EEG studies that investigated the aftereffects of rTMS on EEG measures have already reported substantial discrepancies between EEG-based measures of cortical excitability and MEP amplitude as an index of corticospinal excitability. Van der Werf and Paus [42] applied low-frequency subthreshold rTMS, known to induce depression of MEPs, to the left M1 and did not observe lasting changes in MEP size with respect to the pre-rTMS baseline, while reporting an early reduction followed by a later increase of the amplitude of the N45 TEP component. Similarly, inconsistent results have been obtained after continuous theta burst stimulation (cTBS, [49]). McAllister and colleagues [50] analyzed resting-state EEG over the left M1 before and after cTBS and found that spectral power in the δ, θ, α or β did not predict the MEP reduction after cTBS. Moreover, resting-state EEG power was unaffected by cTBS. Similarly, Vernet and colleagues [51], while reporting that changes in MEP size could be explained by a combination of different TEP peaks at group level, did not report a modulation of specific TEP peaks after cTBS. Another recent cTBS study on motor cortex reported unchanged MEP amplitude and unchanged LMFP over the stimulation site, but decreased δ power in resting-state EEG as well as decreased δ, θ and γ TMS-EEG oscillatory activity after the intervention [52]. Another study [53] did not find any change in TEPs in the presence of a significant increase in MEP size after intermittent theta burst stimulation (iTBS,[49]). Altogether, rTMS interventions thought to induce LTD- or LTP-like changes in corticospinal excitability produced inconsistent effects on all tested readouts, i.e. MEP and EEG measures. In summary, our nil findings are in line with these conflicting results. A possible explanation for these discordant results is that rTMS induces complex changes in molecular and synaptic mechanisms of the cortex that involve both inhibitory and facilitatory effects [54–58]. Since EEG can infer events only on a macroscopic scale and does not discriminate between excitatory and inhibitory processes, rTMS-induced changes in cortical dynamics may remain undetected by the EEG measures used in this study. Moreover, the relationship between MEPs and resting-state or TMS-evoked or induced EEG responses may be not linear, as already suggested by a lack of correlation between these measures in previous studies [12, 50, 52, 59].

A limitation of the present study is the use of suprathreshold TMS intensity in the test blocks pre- and post-rTMS. While this allowed to observe lasting excitability changes on MEPs [15], the associated changes in re-afferent input from the induced muscle twitch could contribute to the modulation of TMS cortical responses. However, the re-afferent input probably did not play a significant role in the reported results, as the investigated EEG measures remained largely unchanged with respect to the pre-rTMS values despite the increase in MEP amplitude. Another limitation is the loss of the EEG signal for up to 15 ms after the TMS pulse. We cannot exclude that significant changes in TMS-evoked or induced EEG responses have occurred in this very early time window, especially because excitation of pyramidal neurons projecting to the spinal cord and responsible for the MEP generation occurs in the very first milliseconds after the TMS pulse [60]. This limitation could be possibly overcome in future studies using faster sampling rates and more focal coils that minimize the activation of scalp muscles and produce smaller TMS related artifacts. Finally, another possible limitation of the study is the use of a data cleaning procedure based on ICA. While ICA is often used and necessary in the analysis of TMS-EEG data [12, 35, 52, 61, 62], it cannot be excluded that the removal of a large number of components partially affected the obtained results.

To conclude, while information over local neuronal excitability state at the time of rTMS has proved to lead to a more controlled efficacy of corticospinal plasticity induction as measured by a LTP-like increase in MEP size (in 91.3% of subjects vs. 52–61% in previous conventional rTMS protocols that did not employ EEG information about ongoing brain state, [15]), we did not observe any consistent modulation of EEG measures and no differential effect dependent on the phase of the ongoing μ-rhythm. The most likely explanation for this nil finding is that resting-state EEG, and TMS-evoked and induced EEG responses do not reflect excitability of corticospinal circuitry.
